# Cerebellar Contributions to Hypokinetic Symptoms in an Acute Lesion Parkinsonism Model

**DOI:** 10.3390/neurolint17050072

**Published:** 2025-05-07

**Authors:** Cristofer Zarate-Calderon, Gerardo Marín, Iraís Viveros-Martínez, Lizbeth Vásquez-Celaya, Porfirio Carrillo-Castilla, Gonzalo E. Aranda-Abreu, Donaji Chi-Castañeda, Luis I. García

**Affiliations:** 1 Instituto de Investigaciones Cerebrales, Universidad Veracruzana, Xalapa 91190, Mexico; 2 Departamento de Neurocirugía, “Hospital Regional 1° de Octubre”, Instituto de Seguridad y Servicios Sociales de los Trabajadores del Estado, Mexico City 07300, Mexico; 3 Instituto de Neuroetología, Universidad Veracruzana, Xalapa 91190, Mexico

**Keywords:** basal ganglia, cerebellum, hypokinetic symptoms, parkinsonism, power spectral density, ventrolateral striatum

## Abstract

**Background:** Parkinsonism, characterized by motor symptoms, is typically attributed to basal ganglia dysfunction. Recent evidence suggests that the cerebellum may also influence these symptoms. This study investigated Crus II, the dentate nucleus (DN), and the inferior olive (IO) in a rat model of parkinsonism induced by a bilateral ventrolateral striatal (VLS) lesion. **Materials and Methods:** Twenty-four male *Wistar* rats were divided into control (*n* = 12) and experimental (*n* = 12) groups. Monopolar electrodes were implanted in target structures. The experimental group received a bilateral VLS lesion. Animals underwent four weekly sessions of electrophysiological recordings and blind behavioral assessments (resting, grooming, locomotion, rearing, sniffing) via video tracking. Power spectral density (PSD) in the 300–500 Hz band was computed. Statistical analyses included Mann–Whitney U, Friedman with Wilcoxon post hoc, and Spearman correlation tests. **Results:** During weeks one and two, there were significant PSD increases in the experimental group compared to the control, particularly in Crus II—grooming (*p* = 0.005), locomotion (*p* = 0.007), and rearing (*p* = 0.026); in IO—sniffing (*p* = 0.0167); and in DN—grooming (*p* < 0.001) and locomotion (*p* = 0.0008). Additionally, intragroup analysis revealed significant PSD elevations relative to baseline in these structures. Significant correlations were observed only for grooming (negative correlations) and sniffing (positive correlations) across all cerebellar regions. **Conclusions:** These findings suggest compensatory cerebellar hyperactivity induced by VLS lesion, potentially modulating hypokinetic symptoms and highlighting dynamic network interactions. Interpretation warrants caution due to limitations inherent to the acute lesion model and experimental duration.

## 1. Introduction

Parkinsonism is a clinical syndrome characterized by motor disturbances, including bradykinesia, muscular rigidity, postural instability, and resting tremors. These features are present, albeit with variable intensity, in a range of parkinsonian syndromes, such as Parkinson’s disease (PD), atypical Parkinsonism (e.g., Lewy body dementia, multiple-system atrophy), and secondary forms resulting from drug induction, vascular lesions, or trauma [1,2,3,4].

Given the heterogeneity inherent to these syndromes, accurate clinical diagnosis requires precise differentiation based on motor and cognitive symptoms [2]. Parkinsonism refers broadly to a syndrome exhibiting shared motor features, whereas PD specifically denotes a progressive α-synucleinopathy characterized by striatonigral degeneration. Crucial diagnostic indicators to differentiate parkinsonian syndromes include responsiveness to levodopa and the severity of motor symptoms, primarily bradykinesia and rigidity, which are not consistently accompanied by resting tremors. Such differentiation is critical because therapeutic strategies and prognostic outcomes vary significantly between PD and other parkinsonian syndromes [3,5].

It is widely established that parkinsonian symptoms primarily result from dysfunction in the nigrostriatal pathway and related motor structures, such as the thalamus and motor cortex, underpinning the therapeutic focus on dopaminergic interventions like levodopa [6]. Recent findings, however, highlight additional cerebral structures that may significantly contribute to the pathophysiology of parkinsonism, influencing symptoms beyond traditional dopaminergic targets, including bradykinesia and tremor [6,7,8].

Although tremors are prominently associated with parkinsonism, hypokinetic symptoms, particularly bradykinesia, and rigidity are essential for accurate diagnosis due to their relative specificity compared to other movement disorders [9,10,11,12]. Importantly, these symptoms manifest differently between PD and secondary or atypical parkinsonism, often being less consistently present in the latter set of conditions [4,13].

Integrating clinical assessments with advanced neurophysiological techniques has significantly advanced our understanding of parkinsonian syndromes, illustrating these conditions as a spectrum of motor dysfunction extending beyond tremors alone [14,15,16].

Animal models, particularly rats, have proven indispensable for elucidating anatomical connectivity and functional roles of neural pathways involved in parkinsonism [17]. Such models enable detailed investigation of parkinsonian features through behavioral and electrophysiological methods [18,19,20]. The ventrolateral striatum (VLS) has become a pivotal target in pharmacological and lesion-induced parkinsonian models [19,21,22].

Recent research emphasizes the functional connectivity between the cerebellum and basal ganglia, suggesting that cerebellar structures like Crus II, the inferior olive (IO), and the dentate nucleus (DN) may contribute significantly to parkinsonian symptomatology through compensatory mechanisms [18,22,23]. Despite extensive research on tremors, there remains a significant gap concerning hypokinetic manifestations such as bradykinesia and rigidity, particularly their associations with cerebellar structures [24,25].

Therefore, this study aimed to investigate cerebellar involvement in both behavioral performance and electrophysiological alterations in a rat model of parkinsonism induced by a mechanical lesion of the VLS, with a special emphasis on hypokinetic symptoms. Based on existing evidence, we hypothesized that VLS lesions alter cerebellar activity, increasing the PSD in Crus II, IO, and DN during tasks requiring greater coordination. Furthermore, we predicted that these changes would be more pronounced in the early post-lesion phase and correlate with observable hypokinetic behaviors, such as prolonged resting periods and shorter episodes of behavioral activity.

## 2. Materials and Methods

### 2.1. Rats and Experimental Groups

Twenty-four male *Wistar* rats (250–350 g, aged postnatal day 60–80) were recruited from the breeding colony at the Instituto de Investigaciones Cerebrales, Universidad Veracruzana. The animals were housed in acrylic cages with sawdust bedding and maintained with *ad libitum* access to food and water. The animal facility followed an inverted 12 h light–dark cycle (lights off from 7:30 to 19:30 h CST). All procedures adhered to the guidelines established in the Official Mexican Standard NOM-062-Z00-1999 [26]. Ethical approval was granted by the Comité Interno para el Cuidado y Uso de Animales de Laboratorio del Centro de Investigaciones Cerebrales (CICUAL-CICE), under approval code 2018-003, dated 1 June 2018.

Animals were divided into control (*n* = 12) and experimental (*n* = 12) groups. To ensure unbiased allocation, a random number generator was used to randomly assign animals to experimental and control groups. Researchers conducting behavioral assessments, electrophysiological recordings, and data analyses were blinded to group assignments, implementing a single-blind design.

Inclusion criteria were *Wistar* rats that met the criteria for sex, weight, and age at the beginning of the experiment and had no previous exposure to experimental or pharmacological procedures. Additionally, animals were required to complete all four scheduled recording sessions without showing signs of illness, discomfort, or surgical complications that could impede the study’s continuation.

Exclusion criteria included rats that did not meet the criteria for sex, weight, and age or had visible signs of illness, injuries, abnormal baseline behavior, infections, electrode displacement, failure to recover from surgery within 72 h, or an inability to complete all four recording sessions due to health deterioration or death during the experimental period.

### 2.2. Electrodes

Given the experimental setup, cerebellar electrical activity was recorded intracranially (in vivo extracellular recording). Two main types of electrodes were used:

1. Recording electrodes consisted of a stainless-steel monopolar bar (diameter: 250 μm, impedance: 3 MΩ) with a length adjusted to the target structure. The electrodes were soldered to male D-Sub crimp pins and insulated with resin, exposing 1 mm of the tip.
2. Reference electrodes consisted of a No. 7 steel screw, 70%-covered with a copper wire (leaving the upper part of the screw exposed). The screw was connected to a male D-Sub crimp pin, with both joints soldered together. It was placed arbitrarily to avoid interference with the recording electrodes.

### 2.3. Surgical Procedures

The stereotaxic neurosurgery procedure has been previously described by Vasquéz-Celaya [22]. All electrode implantations were performed unilaterally in the right hemisphere. The target structures were Crus II, IO, and DN. This procedure was conducted in both groups; however, in the experimental group, prior to electrode implantation at the recording sites, a bilateral craniotomy was performed, followed by the descent of electrodes into the VLS without applying any electrical current. This procedure induced mechanical damage without the need for electrode fixation. The anatomical locations used are listed in Table 1.

Following the procedure, post-surgical care was provided for 72 h before the first recording.

### 2.4. Associated Behaviors

1. Resting: A basal state of inactivity in which the rat maintains a static and relaxed position without exhibiting voluntary or exploratory movements, indicating a low level of motor activation.
2. Grooming: Self-care and hygiene behavior during which the rat uses its limbs, mouth, and tongue to clean and groom different body areas, such as the face, ears, limbs, and fur. This process may also have been associated with emotional state and stress levels.
3. Locomotion (walking): Active horizontal movement involving the voluntary displacement of the rat through the environment. This behavior is characterized by the coordinated use of all four limbs without adopting upright postures or significant changes in body orientation.
4. Rearing: Active vertical exploratory behavior in which the rat elevates itself by supporting its hind limbs, typically extending the forelimbs to examine the surroundings. This behavior is indicative of active environmental stimulus search and spatial evaluation.
5. Sniffing: An exploratory action focused on the sense of smell when resting. The rat performs nasal movements to capture and analyze chemical stimuli in the environment, facilitating food identification, social signal detection, and threat assessment.

Additionally, behavioral and electrophysiological recordings considered some less frequent behaviors, including yawning, scratching (a behavior associated with self-biting in trunk regions, displaying a rhythmic pattern), and mandibular tremors (a parkinsonian behavior characterized by rhythmic jaw movements without an apparent stimulus).

### 2.5. Collection of Data

Following the recovery period, behavioral and electrophysiological activity recordings were conducted. The experimental period consisted of four recording sessions, with the first week (W1) performed immediately after the postoperative period. The second (W2), third (W3), and fourth (W4) weeks were conducted sequentially, with seven-day intervals between each session, for both the control and experimental groups.

Each recording session lasted between 500 and 800 s, during which the five targeted behaviors were documented. Recordings were conducted in a red-light room, isolated mainly from potential auditory, olfactory, or movement disturbances. Before the recording began, and once connected to the recording system, animals were acclimated for 5 min in an acrylic chamber (30 × 30 × 30 cm). After this habituation period, the corresponding recordings were carried out. The recordings were conducted during the same time slot (11:00 to 13:00 h CST). This was carried out to synchronize the animals’ activity patterns with the inverted cycle.

**Figure 1:**
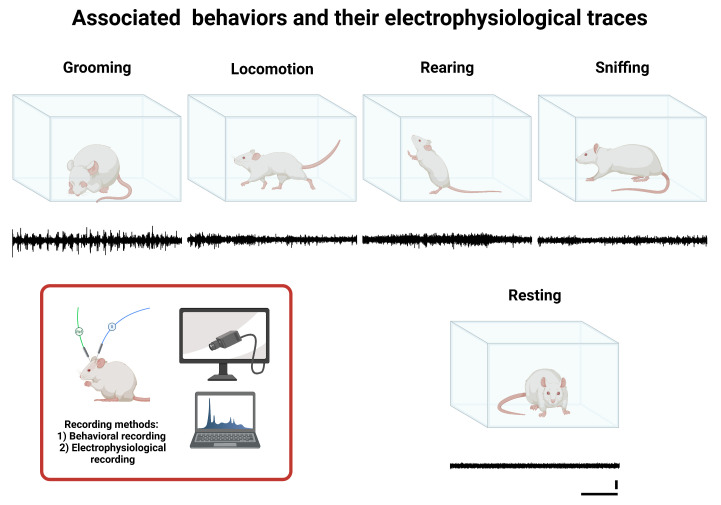
Associated behaviors and their corresponding electrophysiological traces. This figure illustrates study behaviors (Grooming, Locomotion, Rearing, Sniffing, and Resting), and below each behavior’s illustration is a representative example of the corresponding characteristic electrophysiological recording (black line), obtained from the IO via electrode W1. The calibration bars shown under the Resting trace apply to all recordings presented in this figure: the vertical bar represents 1 µV, and the horizontal bar represents 1 s.

A representative diagram of the analyzed behaviors and their characteristic features is shown in Figure 1.

#### 2.5.1. Electrophysiological Recording

Rats were connected to the PolyView 16 system using a 15A54, 15LT amplifier. The amplified signal (amplification: 10; range: 1000; band-pass filter: 100–6000 Hz) was directed to an AM9 audio monitor and a PVA-16 PolyView adapter system, where the signal was digitized (sample rate: 10,000 Hz). The recorded data for each animal were stored using the PolyView Recording System, which allowed for simultaneous annotation and segmentation of observed behaviors in parallel with signal acquisition. This system was manufactured by Grass Technologies, Inc. (West Warwick, RI, USA).

To minimize motion and electromagnetic artifacts, the cables were secured to a low-torque swivel, and the recording area was insulated within a Faraday cage (150 × 150 × 50 cm).

#### 2.5.2. Behavioral Recording

The behavioral recording focused on the timing of the previously described behaviors. Simultaneously with the electrophysiological recording, each behavioral episode’s onset and offset times were documented using an Apple iPad Pro (2nd Generation) video camera (720p resolution at 30 fps) positioned at approximately 40 cm.

Two reviewers subsequently analyzed the videos collected throughout the electrophysiological recording blindly. To refine temporal registration, the timing of each behavior was compared with markers in the electrophysiological recordings. The frequency of occurrence of each behavior throughout the entire observation period was additionally recorded.

The total duration of each behavior was calculated as the sum of the intervals (in seconds) across all recorded episodes.

### 2.6. Electrophysiological Analysis and Power Spectral Density

Five subsequences (“traces”) corresponding to the analyzed behaviors were extracted from each session following the electrophysiological recordings. Each trace was required to be 5 s long, leading to 25 traces per session (corresponding to the five behaviors). Once extracted, the data underwent spectral analysis.

#### Spectral Analysis

The traces were band-pass-filtered (300–3000 Hz, 4th-order Butterworth) with additional notch filtering at 60 Hz and harmonics to eliminate electrical interference. PSD was computed via periodogram and segmented into frequency bands: 300–500 Hz, 500–1000 Hz, 1000–1500 Hz, 1500–2000 Hz, 2000–2500 Hz, and 2500–3000 Hz.

The 300–500 Hz band was analyzed because previous reports indicated this frequency range as physiologically relevant for MUA, representing interneuronal synchronization and fine motor coordination while minimizing electromyographic contamination and electromagnetic artifacts [28,29,30]. Preliminary analyses showing higher normalized PSD (>65% of total power) in this band further supported its selection (Supplementary Material, Figures S1–S3). Traces exhibiting voltage saturation or abrupt excursions exceeding 10 standard deviations were flagged and excluded prior to spectral analysis.

Finally, PSD normalization was performed using the normalized PSD method, defined as follows:

1. Area under the curve (AUC) calculation per frequency band:

Let the frequency band $B_{j}$ be defined by its lower and upper frequency limits, $f_{1}$ and $f_{2}$, respectively. The AUC was computed as follows:

$$AUC_{\left[f_{1},f_{2}\right]}=\sum_{\begin{array}{c} j:\\ f_{1}\leq f_{j}\leq f_{2} \end{array}}PSD\left(f_{j}\right)\;\Delta f_{j}$$

where $PSD\left(f_{j}\right)$ is the power spectral density at frequency $f_{j}$ and $\Delta f_{j}$ is the frequency bin width.

2. Normalization by bandwidth:

The PSD was then normalized by dividing the AUC of each band by its corresponding bandwidth ($B_{j}=f_{2}-f_{1}$):

$$Normalized\;PSD_{\left[f_{1},f_{2}\right]}=\frac{AUC_{\left[f_{1},f_{2}\right]}}{B_{j}}$$

where $B_{j}$ represents the width of the frequency band. This normalization accounts for differences in band size, ensuring that varying bandwidths do not influence power comparisons across bands.

This procedure expresses spectral power density per unit frequency, facilitating unbiased comparisons of relative power across different frequency bands and experimental conditions.

### 2.7. Statistical Analysis

For both analyses, normality and homoscedasticity were assessed using the Lilliefors and Levene tests. Given the sample size and the non-normal distribution of most data, the non-parametric tests were chosen. Brain structures were analyzed separately for spectral analysis.

Therefore, the following statistical approaches were used in the analysis:

- Intergroup comparisons: The Mann–Whitney U test was applied for each measurement week to compare the control and experimental groups. This test was used to identify significant differences in each behavior between the two groups.
- Intragroup comparisons: The Friedman test was employed to evaluate global differences across the different measurement periods within each group. In cases where the Friedman test was significant, *post hoc* analyses were performed using the Wilcoxon tests with Holm adjustment to identify specific differences between weeks.
- Correlation analysis: A correlation analysis was conducted to explore the relationship between time and normalized PSD values. For this, a Spearman correlation test was used. This analysis was performed independently of brain structure and measurement week.

In all analyses, a significance level of *α =* 0.05 was applied. The statistical analyses were conducted using Python 3, employing the *SciPy*, *statsmodels*, and *scikit-posthocs* libraries.

## 3. Results

### 3.1. Intergroup Comparison

**Figure 2:**
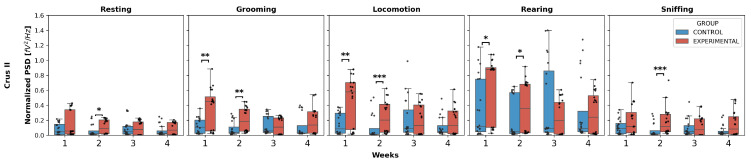
Intergroup comparison of normalized PSD over the weeks for Crus II. This box-and-whisker plot displays the median and interquartile range of the normalized PSD values for behaviors in both groups. Individual black points represent each recorded normalized PSD value. The horizontal axis represents the recording weeks, and the vertical axis shows the normalized PSD values. Asterisks indicate statistically significant differences, with the following thresholds: *p* < 0.05: *; *p* < 0.01: **; *p* < 0.001: ***.

Since the analyses were conducted independently for each structure, the results of the intergroup analysis by structure showed the following:

Crus II (Figure 2): The normalized PSD for resting and sniffing showed statistically significant differences only in W2, where the experimental group exhibited an increase compared to the control group for both behaviors (resting: U = 125.0, *p* = 0.0439; sniffing: U = 70.0, *p* = 0.0005).

Additionally, grooming, locomotion, and rearing showed an increase in weeks 1 and 2 in the experimental group compared to the control group:

- Grooming: W1 (U = 96.0, *p* = 0.0051), W2 (U = 86.0, *p* = 0.0021);
- Locomotion: W1 (U = 100.0, *p* = 0.0071), W2 (U = 73.0, *p* = 0.0006);
- Rearing: W1 (U = 117.0, *p* = 0.0256), W2 (U = 119.0, *p* = 0.0294).

No intergroup differences were found in W3 and W4 for any behavior.

**Figure 3:**
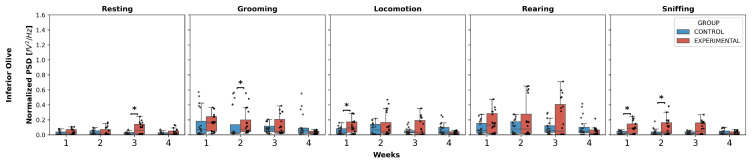
Intergroup comparison of normalized PSD over the weeks for IO. This box-and-whisker plot displays the median and interquartile range of the normalized PSD values for behaviors in both groups. Individual black points represent each recorded normalized PSD value. The horizontal axis represents the recording weeks, and the vertical axis shows the normalized PSD values. Asterisks indicate statistically significant differences, with the following thresholds: *p* < 0.05: *.

Inferior olive (Figure 3): Statistically significant differences were found in resting, specifically an increase in W3 in the experimental group compared to the control (U = 116.0, *p* = 0.0239).

Additionally, grooming showed an increase in normalized PSD in W2 in the experimental group compared to the control (U = 109.0, *p* = 0.0144). Locomotion increased W1 in the experimental group compared to the control (U = 127.0, *p* = 0.0499).

Finally, sniffing exhibited an increase in weeks 1 and 2 in the experimental group compared to the control:

W1 (U = 111.0, *p* = 0.0167);

W2 (U = 118.0, *p* = 0.0275).

Regarding rearing, no significant differences were observed in any week.

**Figure 4:**
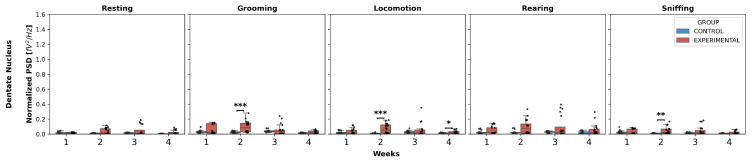
Intergroup comparison of normalized PSD over the weeks for DN. This box-and-whisker plot displays the median and interquartile range of the normalized PSD values for behaviors in both groups. Individual black points represent each recorded normalized PSD value. The horizontal axis represents the recording weeks, and the vertical axis shows the normalized PSD values. Asterisks indicate statistically significant differences, with the following thresholds: *p* < 0.01: **; *p* < 0.001: ***.

Dentate nucleus (Figure 4): Statistically significant differences were found only in W2 in the experimental group compared to the control for the behaviors of grooming, locomotion, and sniffing:

- Grooming: (U = 49.0, *p* < 0.0001);
- Locomotion: (U = 75.5, *p* = 0.0008);
- Sniffing: (U = 99.0, *p* = 0.0066).

No significant differences were found in any week for resting and rearing, nor in W3 and W4 for any behavior.

The median values for the three structures and their respective interquartile ranges are presented in Table A1, Table A2 and Table A3.

### 3.2. Intragroup Comparison

Regarding these comparisons, *post hoc* tests were conducted only for resting, grooming, locomotion, and rearing in Crus II, all behaviors in IO, and resting, grooming, locomotion, and sniffing in DN. These analyses were performed only in the experimental group.

For each behavior in Crus II, post hoc tests revealed the following:

- Resting: W3 and W4 showed a decrease compared to W2.
- Grooming: W3 and W4 showed a decrease compared to W1, while in W3, this decrease was also observed compared to W2, and in W4, it showed an increase compared to W3.
- Locomotion: a decrease was observed in W2, W3, and W4 compared to W1.
- Rearing: W2, W3, and W4 showed a significant decrease compared to W1, a decrease in W3 and W4 compared to W2 was also observed, and W4 showed an increase compared to W3.

Regarding the behaviors in inferior olive, the following was found:

- Resting: W4 represented a decrease compared to W3.
- Grooming: W4 exhibited a significant decrease compared to W1, W2, and W3.
- Locomotion and rearing: a decreasing pattern was observed in W4, but only compared to W1 and W2, with no differences in the remaining weeks.
- Sniffing: W4 decreased compared to W1, W2, and W3, respectively.

Finally, in dentate nucleus, the following was found:

- Resting: there was a decrease only in W4 compared to W3.
- Grooming, locomotion, and sniffing: W4 exhibited a significant decrease compared to W2.

**Figure 5:**
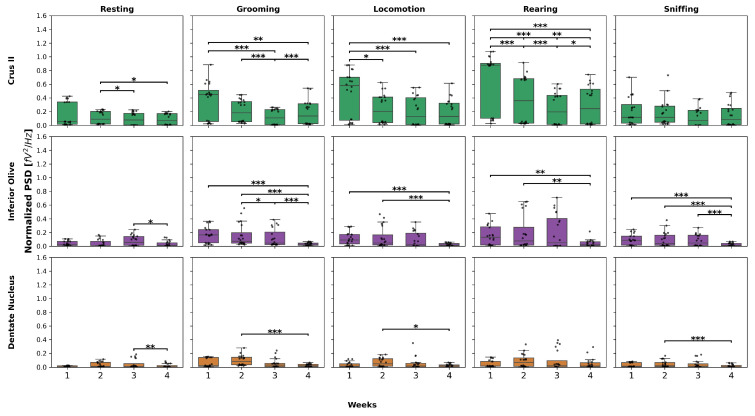
Normalized PSD over the weeks in the experimental group. This box-and-whisker plot displays the median and interquartile range of normalized PSD values for behaviors across the structures in the experimental group. The top graphs correspond to Crus II, the middle to IO, and the bottom to DN. Individual black points represent each recorded normalized PSD value. The horizontal axis represents the recording weeks, while the vertical axis shows the normalized PSD values. Asterisks indicate statistically significant differences, with the following thresholds: *p* < 0.05: *; *p* < 0.01: **; *p* < 0.001: ***.

Figure 5 shows the results highlighted above. The Friedman and Wilcoxon test results for all behaviors and weeks are presented in Table A5, Table A6, Table A7, Table A8, Table A9 and Table A10.

### 3.3. Behavioral Analysis

**Figure 6:**
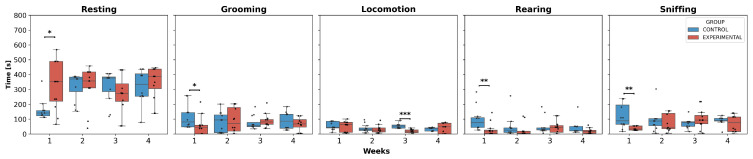
Behavior duration per week. This box-and-whisker plot displays the median values and interquartile ranges for the execution times of behaviors in both groups. Individual black points represent each recorded execution time. The horizontal axis represents the weeks of observation, while the vertical axis indicates the execution time. Asterisks denote statistically significant differences, with *p*-values as *p* < 0.05: *; *p* < 0.01: **; *p* < 0.001: ***.

Regarding the behavioral analyses, only intergroup comparisons were examined, as no significant differences were observed in most weeks for each behavior in the experimental group (Figure 6).

The statistical analysis showed a significant increase in resting time in W1 for the experimental group compared to the control group. In contrast, for grooming, rearing, and sniffing, a significant decrease in the execution time of these behaviors was observed, specifically in W1 for the experimental group compared to the control group. Additionally, locomotion was the only activity that presented significant differences in W3, showing a decreased trend in execution time in the experimental group compared to the control group.

### 3.4. Correlation Analysis

The overall correlation results are shown in Table 2.

Correlations between the behaviors and the normalized PSD were observed; however, statistically significant correlations were found only for grooming and sniffing. In all three evaluated structures, negative correlations were recorded for grooming (Crus II: *p* = 0.0115; IO: *p* = 0.0221; DN: *p* = 0.0070), which implies that longer grooming durations are associated with a decrease in normalized PSD.

Conversely, significant positive correlations were detected for sniffing (Crus II: *p* = 0.0187; IO: *p* = 0.0055; DN: *p* = 0.0187), suggesting that increased normalized PSD accompanies longer sniffing durations. The resting, locomotion, and rearing behaviors did not correlate significantly in any structures.

## 4. Discussion

Cerebellar–BG connections have been extensively studied concerning anatomical and functional aspects; however, their role in movement disorders, specifically parkinsonism, is a growing topic [1,3,7]. While parkinsonism is the most well-known symptomatology of PD, it should not be confused with other movement disorders. Although it can be associated with BG impairment, the involvement of other structures, such as the cerebellum, could broaden the understanding of its motor symptoms and its differentiation from other types of parkinsonian syndromes [4,8,23].

Our results show early cerebellar hyperactivity, characterized by an increased normalized PSD in the 300–500 Hz band due to a VLS lesion compared to the control group, particularly during the early post-lesion phase (W1 and W2), and a decrease in execution time. This activity was consistently observed in the evaluated structures, with greater impact during behaviors that demand higher motor coordination, such as grooming, locomotion, rearing, and sniffing. We justify our focus on the 300–500 Hz band, given that this range is associated with fundamental processes such as interneuronal synchronization, the generation of fine motor patterns, the possible rhythmic organization of motor circuits, and sequence planning [28,30,31,32].

These findings support the hypothesis that the cerebellum acts as a compensatory mechanism against BG dysfunction, a phenomenon consistent with previous reports of alterations in cerebellar activity aimed at mitigating motor deficits after striatal damage [16,33,34]. This initial response could be interpreted as a form of cerebellar “hyperexcitability”, defined by increased neuronal firing rates or greater synchrony, frequently observed after lesions that disrupt the inhibitory–excitatory balance [35,36,37].

Within the experimental group, we noted that, for high coordination behaviors (grooming, locomotion, rearing), the PSD in this band decreased in the last week (W4) compared to the initial weeks, despite general hyperactivity. Although compensatory, this pattern could indicate a specific neuronal reorganization that does not become fully optimized in the early stages of selective dysregulation in cerebellar circuits. Previous studies have described corticostriatal reorganization processes involving cerebellar plasticity to counteract BG deterioration, and our observation of early hyperactivity in W1 and W2, compared to the controls, suggests an activation of these pathways to reinforce motor coordination [23,34,38,39].

In contrast, compared to the intragroup differences, the absence of significant differences in PSD during the later weeks (W3 and W4) within the experimental group points to a self-regulation process. Here, cerebellar activity appears to stabilize as residual neural connections reach a new functional equilibrium, a phenomenon reminiscent of compensatory plasticity that shows transient peaks before converging towards more stable states [34,40,41]. However, this apparent normalization requires cautious interpretation. Experimental factors such as subtle electrode shifts, interindividual variability, circadian cycles, and even tissue changes at the lesion site or around the electrodes could influence late recordings [42,43,44]. Gliosis and alterations at the electrode–tissue interface in chronic implants are known to modify impedance and attenuate MUA signals over time. Although our design, based on relative changes and comparisons with the controls, partially mitigates these effects, the observed dynamics, hyperactivity followed by stabilization, seem to reflect a genuine neurophysiological sequence of compensation and adaptation beyond technical artifacts [42,43,45].

Analyzing the structures individually revealed nuances within the general pattern for PSD. In Crus II, the early increase in PSD in the experimental group compared to control for grooming, locomotion, and rearing suggests compensation in postural motor control and fine movements, given its association with complex motor tasks and cognitive-motor processes [46,47,48]. Meanwhile, in the late phases, the intragroup comparison (decreases in W3/W4) indicates progressive self-regulation, possibly through pathways of synaptic adjustments and cerebellar microcircuit reorganization [32,49,50].

In IO, increases in PSD were reported in the experimental group compared to the control for resting (W3), grooming (W2), locomotion (W1), and sniffing (W1, W2). These increases, particularly during sniffing, could be explained by their connections with the basal ganglia (BG) and contributions from areas such as the zona incerta (sensorimotor integration) [51,52], suggesting an intensification of activation in the early stages during exploratory behaviors or potential stress [53,54]. On the other hand, the decrease in PSD in the intragroup comparison in the late phase (for resting, grooming, and sniffing) supports the existence of compensatory adjustments that reduce initial hyperactivity, aligning with multisystem adaptive plasticity [41,53,55].

Furthermore, in DN, a significant increase in PSD was observed in the experimental group compared to control during the second week (W2) for grooming, locomotion, and sniffing. Given its functional involvement (projections to motor and prefrontal areas) and its described deterioration in PD patients [56,57,58], its early activation in specific motor behaviors supports the hypothesis that the DN triggers a compensatory response due to the VLS lesion. Meanwhile, the decrease in PSD in the intragroup comparison of W4 compared to W2 (for grooming, locomotion, and sniffing) suggests a late reorganization phase, gradually reducing initial overexcitation and promoting a more stable state of activity, reflecting plastic adjustments similar to those described in other cerebellar nuclei [33,34,38,58,59].

Regarding the execution time of behaviors, it was determined that resting significantly increased in the early phase (W1), while grooming, rearing, and sniffing decreased in the same period. There were no changes across the weeks, indicating hypokinetic behavior in the early phase and possible subsequent behavioral adaptability [60,61].

Correlations between behavioral execution and PSD offer additional perspectives. Grooming negatively correlates with PSD (300–500 Hz), highlighting the need to reduce cerebellar activity to synchronize rhythmic/repetitive movements. Conversely, the positive correlation of sniffing with PSD across all structures confirms that olfactory exploration requires greater cerebellar activation, reinforcing its role in both motor and sensory monitoring and postural adjustments [62,63,64]. These behavior-specific changes likely reflect underlying mechanisms: negative correlations with grooming might arise from synaptic down-scaling in Purkinje cell outputs that control rhythmic movements [22,23,65]; positive correlations with sniffing are consistent with the greater coupling of olivo-cerebellar loops during sensorially guided exploratory behaviors [51,52].

These specific patterns could be mediated by circuits such as the dentate–thalamocortical loop, which modulates cortical motor excitability after VLS damage [34,49,66]. Behavioral changes, such as reduced rearing and increased resting in W1, evoke the typical hypokinesia of parkinsonism, suggesting that the cerebellum actively contributes to generating or maintaining these symptoms while compensating for BG damage [40,47,67].

The role of glial cells also warrants attention. Glial cells participate in neural balance restoration post-lesion, modulating neurotransmission, releasing trophic factors, and regulating inflammation, which could influence the observed activity patterns, especially in the deep cerebellar nuclei. This glial component complements the detected neural responses [43,68,69].

While the parkinsonism model induced by the VLS lesion shows the integration of critical cortico-premotor afferents and disynaptic cerebellum–basal-type connections [22,23,70], it has inherent limitations. One of these is that mechanical lesions, although avoiding electrical artifacts, inevitably cause a glial reaction at the lesion site and around the electrode, potentially altering local impedance and MUA amplitudes. Likewise, the mechanical nature does not guarantee absolute specificity; structures adjacent to the VLS are lesioned, possibly including those involved in the observed behavioral patterns.

Clinically, these findings position the cerebellum as a potential therapeutic target, especially in the initial phases of parkinsonism or PD. Modifying deep brain stimulation (DBS) paradigms to include the dentate–thalamic pathway or the DN could complement strategies focused on the BG, reducing symptoms such as gait or balance problems and inducing less sensitivity to dopaminergic treatments or traditional DBS [71,72,73]. Non-invasive techniques, such as transcranial magnetic stimulation (TMS) or transcranial direct current stimulation (tDCS), have already shown promise in this regard [74,75].

From this perspective, the simultaneous evaluation of cerebellar and striatal activity in animal models, combined with neuroimaging (fMRI, tractography), would allow for better characterization of circuit dynamics and anatomical networks [76,77]. This would lead to new therapies that incorporate targeted cerebellar neuromodulation, addressing the pathology from an integrated neural network perspective [38,66,78].

## 5. Perspectives and Limitations

This study opens promising avenues for future research and potential clinical applications. Identifying early cerebellar hyperactivity as a compensatory response to VLS lesions underscores the feasibility of targeting cerebellar structures with greater precision in neuromodulatory therapies. Adapting these interventions by delineating the individual plasticity profiles of cerebellar nuclei may substantially improve therapeutic outcomes in cases of parkinsonism refractory to conventional dopaminergic treatments.

However, certain limitations warrant consideration. The modest sample size and relatively short observation duration might limit the temporal resolution of the compensatory dynamics. Furthermore, a direct assessment of striatocerebellar coupling is lacking; combining VLS electrophysiology with anterograde/retrograde tract-tracing would clarify functional connectivity. Finally, our mechanical approach to VLS lesions affected other structures involved in the observed behaviors. Future investigations with expanded cohorts and prolonged follow-up periods are essential to delineate recovery curves and to better elucidate the long-term functional reorganization within the cerebellar–basal ganglia network.

## 6. Conclusions

The study reveals that mechanical VLS lesions cause early hyperactivity in cerebellar structures (PSD in 300–500 Hz band), with increased resting time and reduced grooming, rearing, and sniffing, suggesting hypokinetic signs or compensation. Later, the PSD decreases, indicating possible adaptation. Correlations are observed between PSD and behaviors (negative with grooming, positive with sniffing), suggesting differential cerebellar modulation. This highlights the cerebellum–basal ganglia interaction and suggests cerebellar nodes as neuromodulation targets for parkinsonism, although it is limited by the acute lesion model, the short timeframe, and the lack of connectivity data.

## Figures and Tables

**Table 1:** Coordinates of the structures. This table presents the coordinates used in the present study, with references from the Bregma point. The values were obtained from *The Rat Brain in Stereotaxic Coordinates*. AP: anteroposterior; ML: mediolateral; DV: dorsoventral [27].

Structure	AP [mm]	ML [mm]	DV [mm]
VLS	−0.48	±4.40	−6.80
Crus II	−14.00	3.40	−5.00
IO	−11.80	0.80	−11.00
DN	−11.30	3.40	−6.40

**Table 2:** Correlation between time and normalized PSD. This table presents the overall results of Spearman correlations between normalized PSD and behavior execution time.

Behavior	Structure		
	Crus II	IO	DN
Resting	−0.038235294	−0.05	−0.241176471
Grooming	−0.6136867	−0.566593237	−0.644591786
Locomotion	0.380532629	0.286136938	0.333334784
Rearing	0.085546095	0.209440439	0.292036669
Sniffing	0.579411765	0.658823529	0.579411765

## Data Availability

The text fully presents all results from the statistical tests, including median values. Readers requiring access to the underlying dataset for further analysis or verification may contact the corresponding author.

## Supplementary Materials

The following supporting information can be downloaded at https://www.mdpi.com/article/10.3390/neurolint17050072/s1: Figure S1: Evaluation of the average normalized PSD in Crus II; Figure S2: Evaluation of the average normalized PSD in IO; Figure S3: Evaluation of the average normalized PSD in DN.

## Abbreviations

The following abbreviations are used in this manuscript:

AP	Anteroposterior
AUC	Area Under the Curve
BG	Basal Ganglia
DBS	Deep Brain Stimulation
DN	Dentate Nucleus
DV	Dorsoventral
IO	Inferior Olive
ML	Mediolateral
MUA	Multiunit Activity
PD	Parkinson’s Disease
PSD	Power Spectral Density
VLS	Ventrolateral Striatum

## Appendix A

### Appendix A.1. Median Values of Normalized PSD and Behavior Execution Time

This section presents the key values for each structure. Table A1, Table A2 and Table A3 display the corresponding normalized PSD values for the structures, while Table A4 presents the median and interquartile ranges of behavior execution time.

**Table A1:** Normalized PSD values for Crus II. This table presents the calculated median values and interquartile ranges for the normalized PSD over the weeks and the analyzed activities in both groups for Crus II.

Structure	Behavior	Week	Control [fV^2^/Hz]			Experimental [fV^2^/Hz]		
			Median	Q1	Q3	Median	Q1	Q3
Crus II	Resting	1	0.0460	0.0079	0.1452	0.0543	0.0158	0.3390
		2	0.0187	0.0111	0.0579	0.0895	0.0195	0.2038
		3	0.0463	0.0159	0.1181	0.0775	0.0025	0.1758
		4	0.0241	0.0165	0.0569	0.0696	0.0081	0.1704
	Grooming	1	0.1062	0.0245	0.2015	0.4488	0.0568	0.5124
		2	0.0399	0.0231	0.1105	0.1817	0.0533	0.3471
		3	0.0572	0.0371	0.2513	0.1072	0.0100	0.2297
		4	0.0304	0.0236	0.1269	0.1352	0.0247	0.3144
	Locomotion	1	0.0844	0.0258	0.2938	0.5793	0.0738	0.7022
		2	0.0136	0.0093	0.0901	0.2016	0.0404	0.4122
		3	0.0870	0.0394	0.3372	0.1269	0.0112	0.4024
		4	0.0437	0.0356	0.1270	0.1301	0.0204	0.3227
	Rearing	1	0.1192	0.0482	0.7493	0.8787	0.1040	0.9025
		2	0.0455	0.0223	0.5684	0.3583	0.0314	0.6795
		3	0.0926	0.0389	0.8566	0.1967	0.0132	0.4365
		4	0.0864	0.0456	0.3204	0.2417	0.0197	0.5270
	Sniffing	1	0.0907	0.0206	0.1563	0.1171	0.0321	0.3034
		2	0.0163	0.0117	0.0611	0.1168	0.0448	0.2793
		3	0.0478	0.0183	0.1281	0.0730	0.0020	0.2184
		4	0.0289	0.0182	0.0533	0.0831	0.0115	0.2468

**Table A2:** Normalized PSD values for IO. This table presents the calculated median values and interquartile ranges for the normalized PSD over the weeks and the analyzed activities in both groups for IO.

Structure	Behavior	Week	Control [fV^2^/Hz]			Experimental [fV^2^/Hz]		
			Median	Q1	Q3	Median	Q1	Q3
Inferior olive	Resting	1	0.00969	0.00404	0.0382	0.026	0.01	0.0687
		2	0.0218	0.0088	0.0522	0.00969	0.0084	0.0694
		3	0.0115	0.00908	0.0307	0.0564	0.0117	0.14166
		4	0.012	0.00478	0.029	0.0117	0.00264	0.05
	Grooming	1	0.0685	0.0177	0.18222	0.164119	0.0493	0.241812
		2	0.0246	0.0203	0.135983	0.0717	0.0438	0.197312
		3	0.0758	0.0412	0.116298	0.0473	0.0242	0.205878
		4	0.0588	0.0207	0.0888	0.0293	0.0137	0.0469
	Locomotion	1	0.0355	0.0124	0.0821	0.0912	0.0343	0.170791
		2	0.0649	0.0082	0.139841	0.0464	0.0194	0.16514
		3	0.0334	0.0259	0.0544	0.0271	0.00386	0.190242
		4	0.0365	0.013	0.0973	0.0159	0.00768	0.0398
	Rearing	1	0.0591	0.0229	0.151807	0.129852	0.018	0.282026
		2	0.0767	0.0199	0.174694	0.0775	0.0176	0.275724
		3	0.0593	0.0327	0.11991	0.0491	0.00321	0.404475
		4	0.0435	0.0248	0.102217	0.0211	0.00745	0.0644
	Sniffing	1	0.0193	0.0123	0.0482	0.0865	0.0156	0.146023
		2	0.0162	0.00815	0.0379	0.0382	0.0151	0.160667
		3	0.0206	0.011	0.0409	0.0468	0.00948	0.159506
		4	0.0203	0.00933	0.0486	0.0145	0.00375	0.0417

**Table A3:** Normalized PSD values for DN. This table presents the calculated median values and interquartile ranges for the normalized PSD over the weeks and the analyzed activities in both groups for DN.

Structure	Behavior	Week	Control [fV^2^/Hz]			Experimental [fV^2^/Hz]		
			Median	Q1	Q3	Median	Q1	Q3
Dentate nucleus	Resting	1	0.00636	0.00394	0.0189	0.00691	0.00584	0.021
		2	0.007	0.00506	0.0119	0.0117	0.00544	0.0698
		3	0.00642	0.00334	0.0192	0.00934	0.0022	0.0507
		4	0.00479	0.00319	0.00528	0.00615	0.00167	0.0226
	Grooming	1	0.0219	0.0169	0.0319	0.0238	0.0115	0.139797
		2	0.0209	0.014	0.029	0.0825	0.0341	0.14262
		3	0.0354	0.0285	0.0447	0.0296	0.00697	0.0554
		4	0.00964	0.00741	0.0179	0.0217	0.00805	0.0407
	Locomotion	1	0.01	0.0086	0.0224	0.0123	0.00832	0.0485
		2	0.00866	0.00701	0.0106	0.0469	0.0126	0.123047
		3	0.0219	0.0102	0.0317	0.0155	0.00246	0.0544
		4	0.00602	0.00484	0.0158	0.0246	0.00516	0.0322
	Rearing	1	0.0177	0.0129	0.0231	0.0164	0.0136	0.0823
		2	0.0175	0.0134	0.0198	0.0681	0.0104	0.13338
		3	0.0294	0.0204	0.0326	0.02	0.00276	0.0952
		4	0.00811	0.00538	0.0288	0.0163	0.00377	0.0638
	Sniffing	1	0.018	0.00971	0.0262	0.012	0.00914	0.0667
		2	0.00878	0.00669	0.0113	0.0214	0.00944	0.0668
		3	0.0153	0.00733	0.0182	0.0126	0.00177	0.0472
		4	0.00657	0.00387	0.0116	0.00616	0.00232	0.0257

**Table A4:** Behavior execution time. This table presents the calculated median values and interquartile ranges for the behavior execution time over the weeks and the analyzed activities in both groups.

Behavior	Week	Control [s]			Experimental [s]		
		Median	Q1	Q3	Median	Q1	Q3
Resting	1	141.15	122.23	157.90	352.80	221.68	488.50
	2	370.70	285.76	383.15	356.95	309.80	417.40
	3	377.05	285.35	384.26	273.55	219.68	338.51
	4	334.92	253.10	404.83	387.70	305.84	437.00
Grooming	1	80.53	47.45	144.60	43.23	0.00	59.00
	2	94.25	9.00	127.95	71.00	18.90	177.85
	3	55.72	49.15	77.73	81.10	65.25	97.88
	4	86.98	38.54	132.46	66.80	45.69	96.45
Locomotion	1	44.70	40.41	85.04	62.60	10.60	78.05
	2	30.00	22.96	40.46	25.70	14.23	40.05
	3	52.55	39.39	60.55	18.58	8.59	27.45
	4	38.40	18.70	40.60	48.60	0.00	72.00
Rearing	1	75.30	43.70	109.53	19.65	3.20	26.11
	2	25.43	9.53	40.73	3.70	2.55	18.40
	3	27.30	27.30	40.60	45.25	11.63	56.64
	4	20.90	20.45	51.40	15.40	3.30	24.55
Sniffing	1	88.05	65.43	194.66	33.20	25.54	53.95
	2	87.25	56.30	102.50	43.50	34.60	138.31
	3	76.63	52.05	82.60	93.25	68.60	124.55
	4	88.95	87.00	104.30	75.90	16.00	114.56

### Appendix A.2. PSD Statistical Test Values for Intragroup Comparison

This section presents the statistical results of the Friedman and Wilcoxon tests with Holm adjustment for the intragroup analysis. Table A5, Table A6, Table A7, Table A8, Table A9 and Table A10 display these results solely for the experimental group.

**Table A5:** Statistical values for Crus II. This table shows the statistical values obtained from the Friedman and Wilcoxon tests for comparisons between weeks in Crus II.

Structure	Behavior	Friedman Statistical Value	Wilcoxon Statistical Value by Weeks Compared					
			(1, 2)	(1, 3)	(1, 4)	(2, 3)	(2, 4)	(3, 4)
Crus II	Resting	18.78	87	56	56	21	16	102
	Grooming	48.48	28	0	10	0	37	0
	Locomotion	29.70	16	0	4.5	54	50	92
	Rearing	54.18	1	0	0	0	11	18
	Sniffing	15.18	102	50	64	56	65	57

**Table A6:** *p*-Values for Crus II. This table shows the *p*-values obtained from the Friedman and Wilcoxon tests with Holm adjustment for the comparisons between weeks in Crus II.

Structure	Behavior	Friedman *p*-Value	Wilcoxon *p*-Value by Weeks Compared					
			(1, 2)	(1, 3)	(1, 4)	(2, 3)	(2, 4)	(3, 4)
Crus II	Resting	0.0003036	1.0	1.0	1.0	0.0280724	0.0116043	1.0
	Grooming	0.0000000	0.0840797	0.0000916	0.0031986	0.0000916	0.2641983	0.0000916
	Locomotion	0.0000016	0.0116043	0.0000916	0.0007629	1.0	0.8797684	1.0
	Rearing	0.0000000	0.0001564	0.0000916	0.0000916	0.0000916	0.0039864	0.0164070
	Sniffing	0.0016691	1.0	0.8797684	1.0	1.0	1.0	1.0

**Table A7:** Statistical values for IO. This table shows the statistical values obtained from the Friedman and Wilcoxon tests for the comparisons between weeks in IO.

Structure	Behavior	Friedman Statistical Value	Wilcoxon Statistical Value by Weeks Compared					
			(1, 2)	(1, 3)	(1, 4)	(2, 3)	(2, 4)	(3, 4)
Inferior olive	Resting	21.30	93	43	88	43	48	23
	Grooming	42.54	92	52	0	19	0	0
	Locomotion	27.42	93	75	5	54	0	57
	Rearing	23.40	69	87	10	73	13	53
	Sniffing	34.90	86	96	0	52	1	0

**Table A8:** *p*-Values for IO. This table shows the statistical values obtained from the Friedman and Wilcoxon tests for the comparisons between weeks in IO.

Structure	Behavior	Friedman *p*-Value	Wilcoxon *p*-Value by Weeks Compared					
			(1, 2)	(1, 3)	(1, 4)	(2, 3)	(2, 4)	(3, 4)
Inferior olive	Resting	0.0000912	1.0	0.4423752	1.0	0.4423752	0.6553650	0.0302315
	Grooming	0.0000000	1.0	0.9203777	0.0000687	0.0152245	0.0000687	0.0000687
	Locomotion	0.0000048	1.0	1.0	0.0005531	0.9903870	0.0000687	1.0
	Rearing	0.0000333	1.0	1.0	0.0022964	1.0	0.0045319	0.9570465
	Sniffing	0.0000001	1.0	1.0	0.0000687	1.0	0.0001144	0.0000687

**Table A9:** Statistical values for DN. This table shows the statistical values obtained from the Friedman and Wilcoxon tests for comparisons between weeks in DN.

Structure	Behavior	Friedman Statistical Value	Wilcoxon Statistical Value by Weeks Compared					
			(1, 2)	(1, 3)	(1, 4)	(2, 3)	(2, 4)	(3, 4)
Dentate nucleus	Resting	12.12	59	65	72	87	42	9
	Grooming	22.02	60	104	54.5	25	0	35
	Locomotion	13.14	56	93	97	49	17	81
	Rearing	12.78	51	60	96	70	31	87
	Sniffing	20.22	62	83	36	52	0	51

**Table A10:** *p*-Values for DN. This table shows the statistical values obtained from the Friedman and Wilcoxon tests for comparisons between weeks in DN.

Structure	Behavior	Friedman *p*-Value	Wilcoxon *p*-Value by Weeks Compared					
			(1, 2)	(1, 3)	(1, 4)	(2, 3)	(2, 4)	(3, 4)
Dentate nucleus	Resting	0.0069832	1.0	1.0	1.0	1.0	0.5841675	0.0032101
	Grooming	0.0000646	1.0	1.0	1.0	0.0692863	0.0001030	0.2626419
	Locomotion	0.0043434	1.0	1.0	1.0	1.0	0.0185566	1.0
	Rearing	0.0051373	1.0	1.0	1.0	1.0	0.1603966	1.0
	Sniffing	0.0001528	1.0	1.0	0.2907944	1.0	0.0001030	1.0
