# Supplementary material for: Cerebellar Contributions to Hypokinetic Symptoms in an Acute Lesion Parkinsonism Model
Source: Neurol Int. 2025 May 7;17(5):72. doi: 10.3390/neurolint17050072 (PMC12113700; doi:10.3390/neurolint17050072)
Supplement: Supplementary file 1 [file neurolint-17-00072-s001.zip › neurolint-3569524-supplementary.pdf]

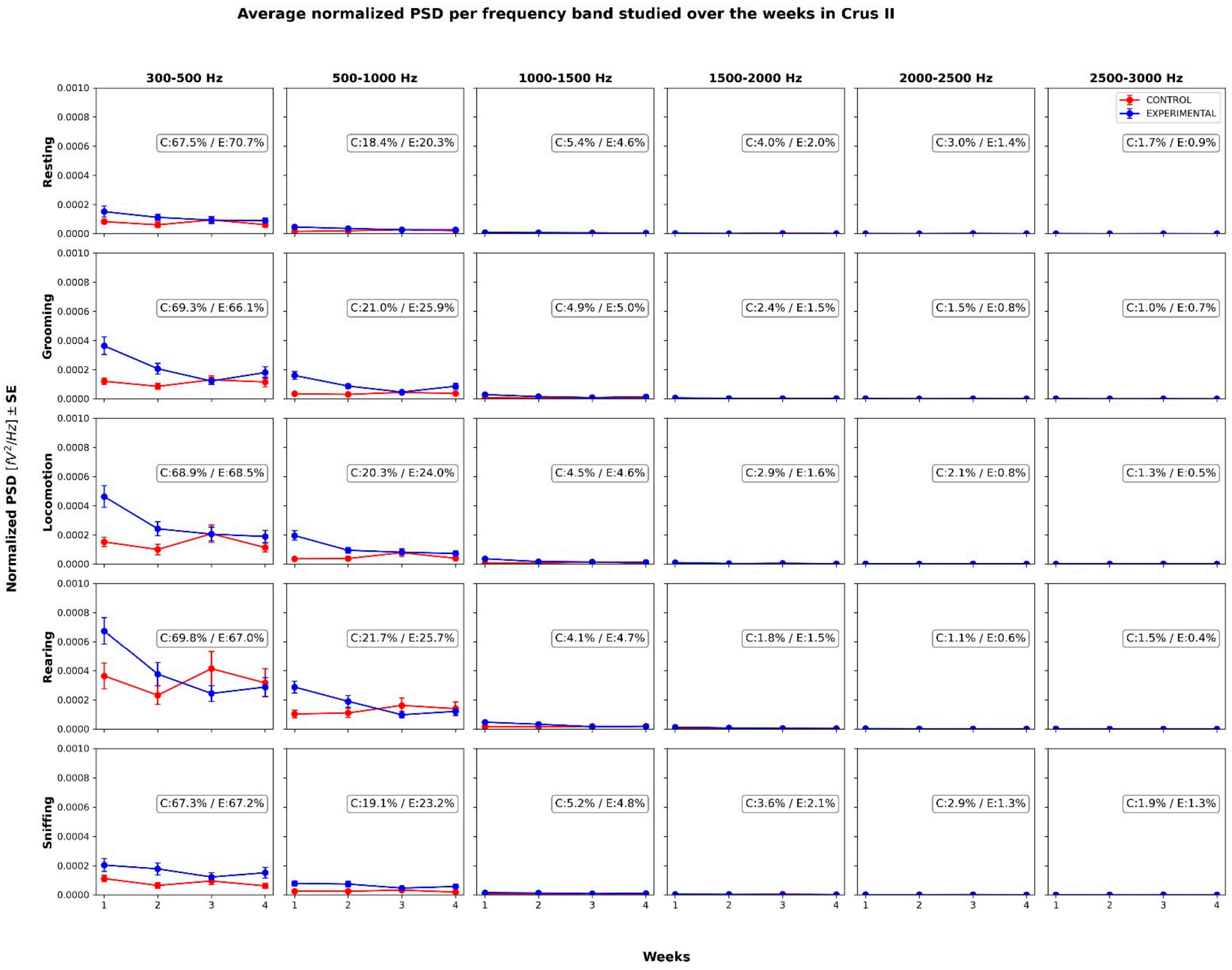

Figure S1. Evaluation of the average normalized PSD in Crus II**.** Mean values ± standard error are plotted over the weeks for the five analyzed behaviors across their respective frequency bands in Crus II. The X-axis represents weeks, and the Y-axis represents PSD values. Insets in each plot indicate the average percentage of PSD per band and group (C: control, E: experimental).

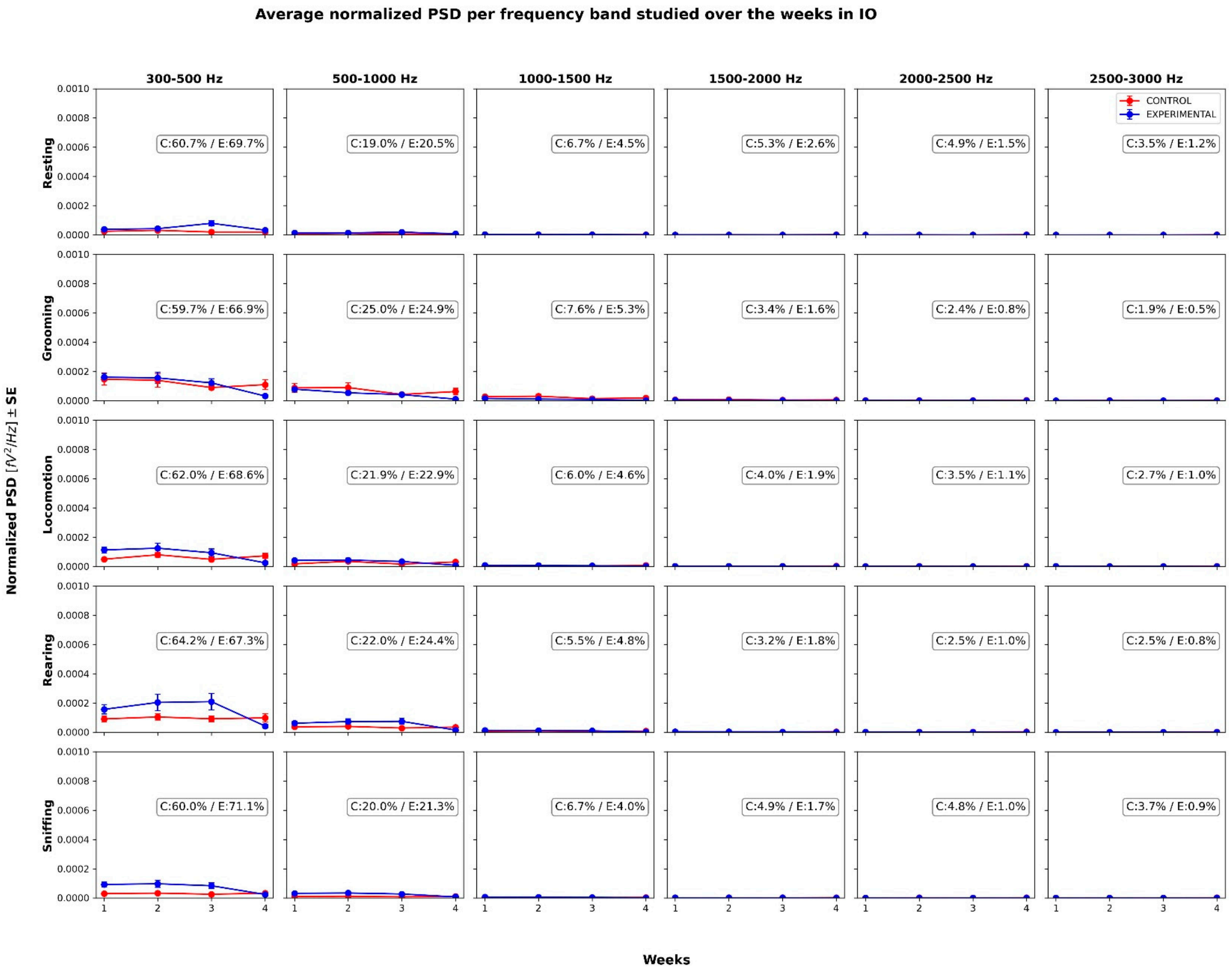

Figure S2. Evaluation of the average normalized PSD in IO. Mean values ± standard error are plotted over the weeks for the five analyzed behaviors across their respective frequency bands in IO. The X-axis represents weeks, and the Y-axis represents PSD values. Insets in each plot indicate the average percentage of PSD per band and group (C: control, E: experimental).

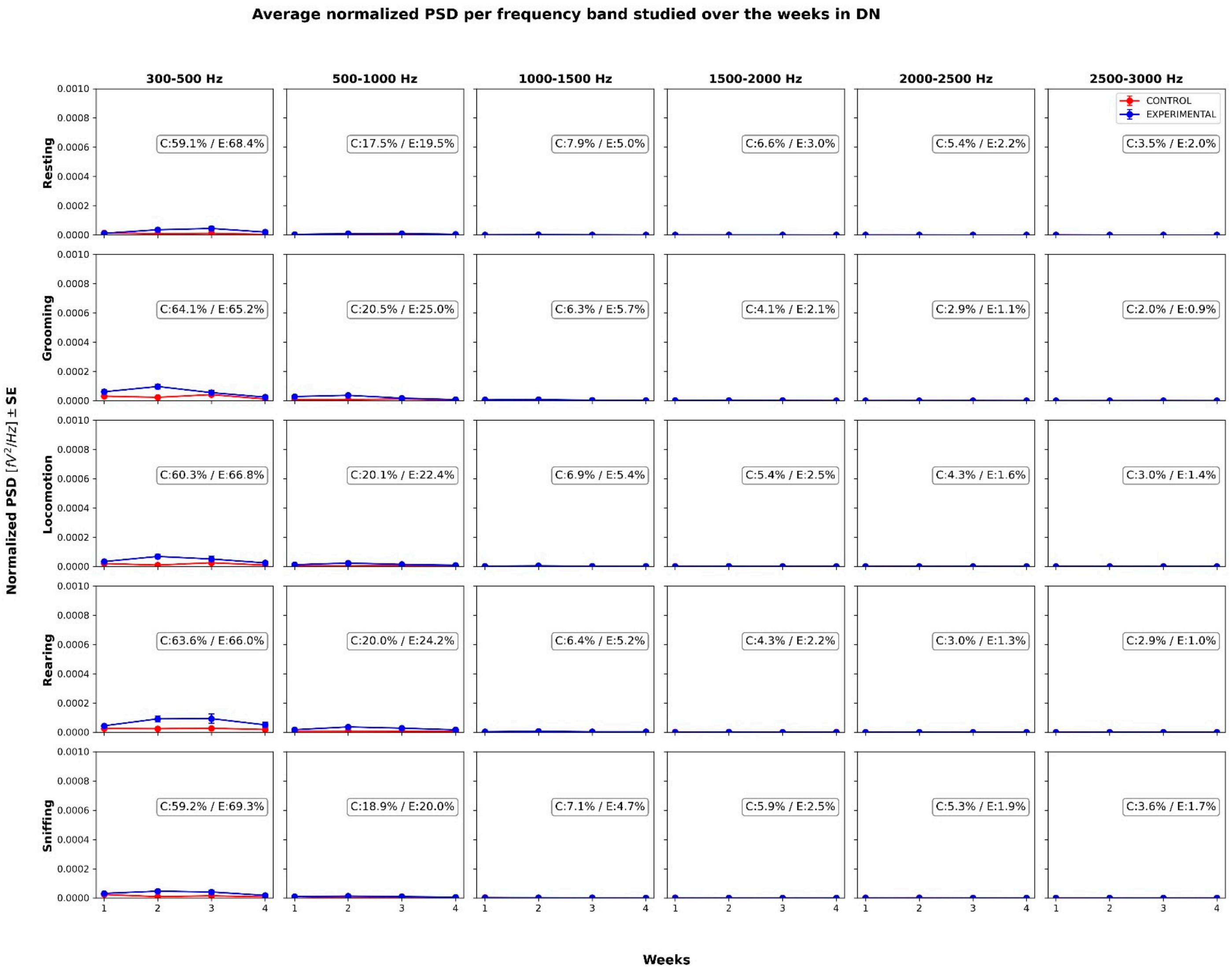

Figure S3. Evaluation of the average normalized PSD in DN. Mean values ± standard error are plotted over the weeks for the five analyzed behaviors across their respective frequency bands in DN. The X-axis represents weeks, and the Y-axis represents PSD values. Insets in each plot indicate the average percentage of PSD per band and group (C: control, E: experimental).
